# Reflections of Graduating Medical Students: A Photo-Elicitation Study

**DOI:** 10.1007/s40670-023-01758-3

**Published:** 2023-02-16

**Authors:** Robert Wilson, Karan Varshney, Matteo Petrera, Nathan Hoff, Vanessa Thiel, Rosemary Frasso

**Affiliations:** 1 College of Population Health, Thomas Jefferson University, Philadelphia, USA; 2 Sidney Kimmel Medical College, Thomas Jefferson University, Philadelphia, USA; 3 Deakin School of Medicine, Deakin University, Geelong, Australia

**Keywords:** Medical students, Photovoice, Photo-elicitation, Burnout, Coping, Frustration, Wellness

## Abstract

Medical school is associated with increased mental health morbidity that can result in professional burnout. To explore the sources of stress and means of coping for medical students, the photo-elicitation method was utilized, with interviews being conducted. The commonly discussed stressors included the presence of academic stress, difficulty relating to peers outside of medicine, frustration, feelings of helplessness and under-preparedness, imposter syndrome, and competition. Coping themes included camaraderie, interpersonal relationships, and wellness activities such as diet and exercise. Medical students are exposed to unique stressors, and as a result, students develop coping strategies throughout their studies. Further research is needed to identify how to better support students.

**Supplementary Information:**

The online version contains supplementary material available at 10.1007/s40670-023-01758-3.

## Introduction

Burnout is a well-documented phenomenon affecting almost half of US practicing physicians [1, 2]. It is not only detrimental to providers, but it has also been linked to increased adverse events and poorer patient outcomes [2]. Research suggests that the burnout process oftentimes begins in medical school [3, 4]. As such, administrators have implemented various strategies to prevent burnout, including mental health services to curricula changes [3, 4]. Questions remain, however, about the causes of burnout, and the role of medical school in the phenomenon is not fully understood. Photo-elicitation has been used in previous studies to understand lived realities of students in medical school and other educational settings [5, 6]. However, these studies have largely focused strictly on the impacts of COVID-19; to our knowledge, the photo-elicitation methodology has not previously been used to understand sources of stress, and means of coping, across the duration of the medical school experience. Therefore, we conducted a qualitative study using photo-elicitation to shed light on stressors and coping strategies of medical students, and to identify early opportunities to address burnout.

## Activities

This study took place during the 2019–2020 academic year at Thomas Jefferson University under the approval of the Institutional Review Board. A research team of medical students (RW, NT, MP, RT) was trained to conduct photo-elicitation interviews; that is, to use participant-generated images as a guide [7, 8]. Participants were then recruited from the Sidney Kimmel Medical College graduating class using a combination of convenience and snowball sampling methodologies.

Each participant attended two video meetings with a member of the research team (RW, MP, RT, NH). During the first meeting, a researcher explained the study, obtained informed consent, and reviewed the ethical use of photography in research [7, 8]. Additionally, the participants were instructed to take photographs or gather old photographs that would shed light on their stressors and means of addressing stress during medical school. While data collection did occur during the start of the COVID-19 pandemic, participants were instructed to provide images that were representative of their hardships across their entire duration of medical school. They were given roughly one week to submit these photographs, and they were instructed to exclude any photographs of illegal activity or those that contained patient information.

During the second meeting, participants shared their photographs with a member of the research team and completed an interview. The researcher began by asking an open-ended question, such as “tell me about this photograph.” Follow-up questions were used to further explore themes and obtain clarification to help understand the significance of the photographs.

All interviews were audio-recorded, transcribed, and analyzed using NVivo12 software. Interview transcriptions were independently coded by two members of the research team (RW, KV). The coders met and resolved all coding discrepancies. Codes were organized into two primary groups based on the general principle of stress and coping described by Lazarus and Folkman: sources of perceived stress and methods of coping with these stressors [9]. Using a directed-content analysis, a priori thematic categories were identified from previous research on medical student well-being. Other themes that arose during analysis were added [10]. The codebook that was utilized is shown in Supplementary Table 1.

## Results

Ten students shared photographs and participated in the interviews. All students were in the process of completing their final year of medical school at Sidney Kimmel Medical College in Philadelphia, Pennsylvania. Nine interview transcripts were ultimately analyzed (due to technical difficulties with one participant’s recorded interview); there were two males and seven female participants and a combined total of 66 photos collected. Intercoder reliability was assessed, and the team achieved reliability (*k*) > 0.8. Recurring thematic categories fell within the frameworks of stress and coping.

**Fig. 1:**
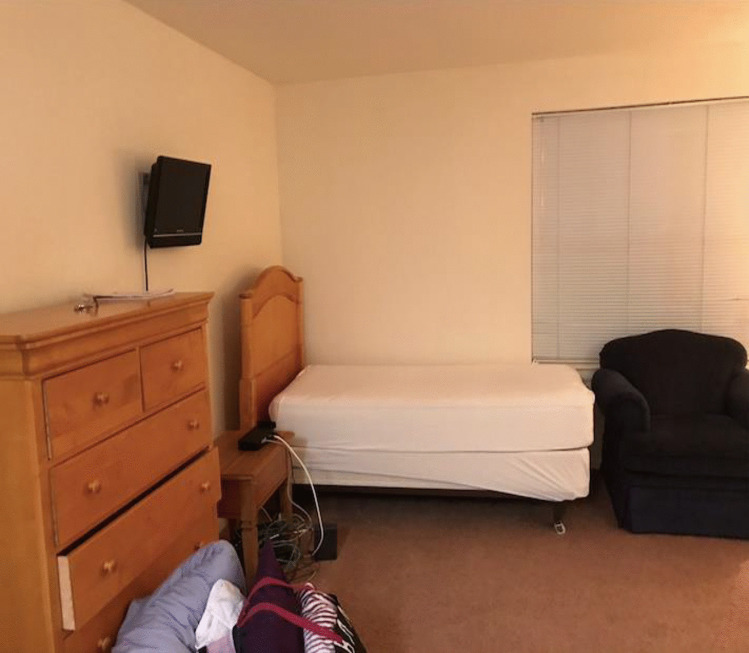
A student submitted a photograph of an empty dormitory room and described the isolation he/she/they experienced throughout medical school

Content analysis revealed that feeling disconnected/isolated, having imposter syndrome/comparisons with others, stress from board exams, COVID-19, and identity not feeling understood were among the most commonly identified sources of stress. On feeling isolated, one participant submitted a photo of an empty dormitory room and explained the photo, saying “We’re kind of isolated from people… whether we’re on a particular [away] rotation where we’re physically isolated from people or mentally isolated… the whole process can really just kind of bring us apart from people” (Fig. 1). Other perceived stressors included loss of previous self-identity, mental/physical exhaustion, academic stress, feelings of helplessness, competition, and a sense of under-preparedness. Frustration was denoted as a result of stressors. Table 1 lists frequencies of themes described regarding stressors and strategies to face stress, respectively.

**Table 1:** Stressors and strategies to deal with stressors among participants

**Stressors throughout medical school (*n*** **= 350)**	***n*** **(%)**	**Strategies to deal with stress (*n*** **= 192)**	***n*** **(%)**
Stress (overwhelmed/stressed/anxious/helpless/fearful)	26 (7.4)	Coping (how/strategy described)	36 (18.8)
Development of cynicism/loss of idealism	28 (8.0)	Interpersonal relationships	42 (21.9)
Under-preparedness/gaps in education	21 (6.0)	Support	31 (16.1)
Hierarchy	9 (2.6)	Sleep/diet/exercise	13 (6.8)
Evaluation	10 (2.9)	Camaraderie	34 (17.7)
Imposter syndrome/comparison with others	13 (3.7)	Compartmentalization	10 (5.2)
COVID-19	37 (10.6)	Extra-curricular activities	14 (7.3)
Board exams	32 (9.1)	Development of substance use/unhealthy behaviors	10 (5.2)
Disconnected/isolated	34 (9.7)	Mental health treatment/therapy	2 (1.0)
On-hold/loss of interest in hobbies	26 (7.4)		
Uselessness/not knowing enough	24 (6.9)		
Competition	10 (2.9)		
Anatomy	10 (2.9)		
Loss of identity/individualism	20 (5.7)		
Identity not understood (by others)	34 (9.7)		
Exhaustion	16 (4.6)		

**Fig. 2:**
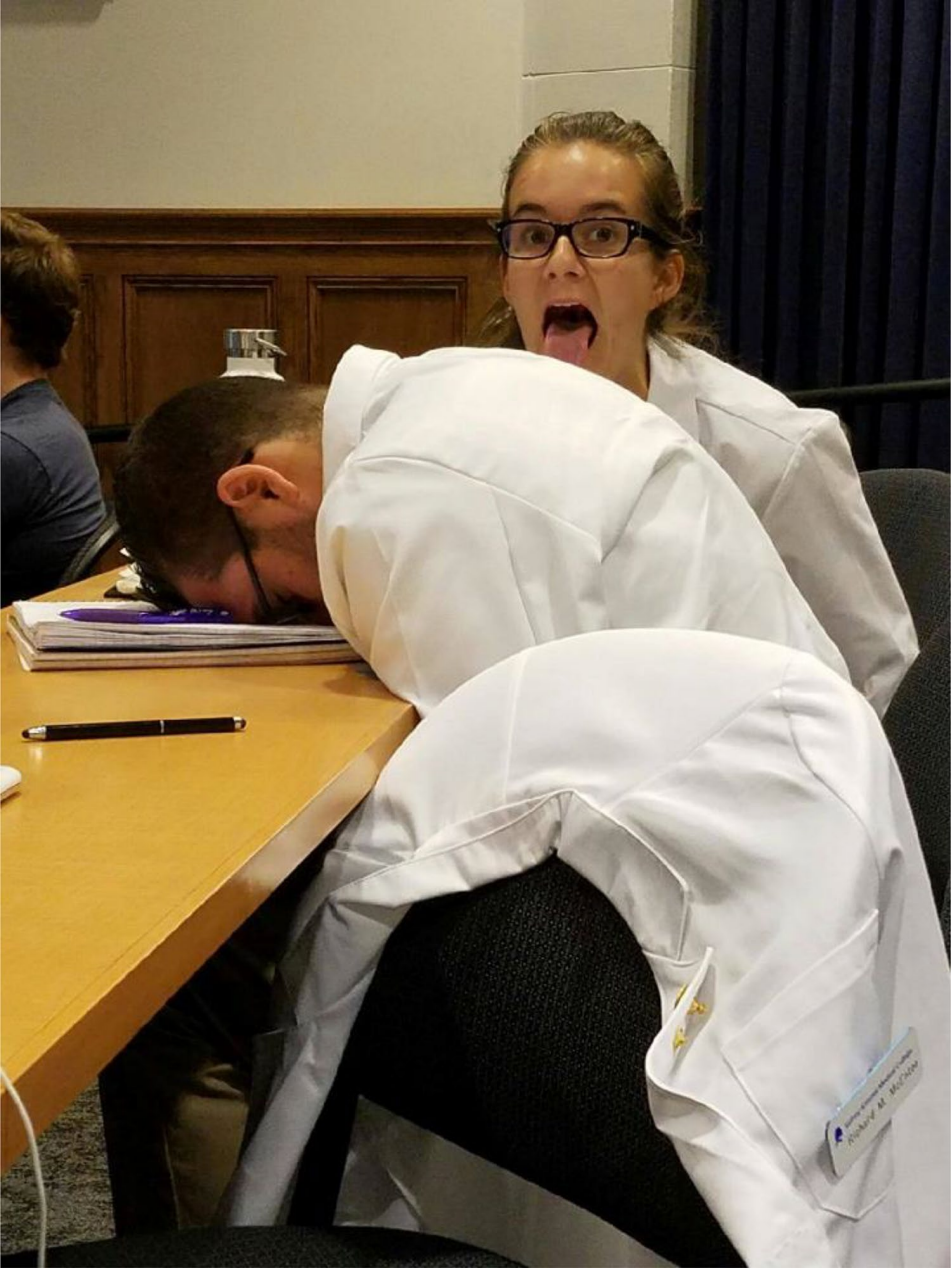
A student submitted photograph of two students joking with one another during a lecture and described camaraderie as a coping strategy

Student-identified coping strategies included those that were effective, such as camaraderie, exercise, diet, and sleep. Interpersonal relationships were identified as important in helping students coping and the most common strategy/means to deal with stress. Reflecting on a photograph of two students making funny faces during a lecture, one participant said “…I guess medical students are kind of it in it together, even when it’s so s****y and you have all this s**t to learn, there’s still time in every day to take a funny photo with your friends and have some laughs” (Fig. 2). Less effective coping strategies, such as alcohol misuse and development of cynical outlook on career and training, were also described among participants.

Some content did not fall into traditional frameworks of stress and coping. One example came from a student who described their sacrifices made while in medical school. The student denoted that numerous dreams, goals, and aspirations outside of medicine had to be sacrificed in order to be able to succeed in medical school. Another pertinent example of content falling outside the traditional frameworks came from one student who reflected on the usage of medications while in medical school. One student, who shared a photograph of his/her anti-depressant medication, reflected by stating that “It’s almost like each pill is a reminder that I wasn’t strong enough to be the perfect med student… Many of my friends in med school are on similar meds, so I find solace in that, but I feel like we’re all sharing this dirty secret together. I can’t tell if I’m happy that med school unearthed this underlying pathology that I never fully was aware of, or angry that school broke me down to this point.”

## Discussion

While a growing body of research demonstrates increasing prevalence of burnout among medical students and trainees, the basis for this phenomenon is not entirely understood. Our study explored the potential foundations for these concerning trends in a qualitative, participant-directed, photo-elicitation manner.

As our study was qualitative, specific trends cannot be gleaned from our findings. However, our findings indicate that burnout and career dissatisfaction could result from a variety of stressors that begin to surface early in training and are unique to medical students and may rely to some degree on their ability to use effective coping mechanisms. That is, individual students’ backgrounds, personality traits, and performance might result in different stressors and coping strategies. As a result, “one-size-fits-all” approaches to fostering wellness and reducing burnout among medical students are likely overly simplistic and inadequate.

Our study brings words and images to light that describe the realities for medical students within the broader context of wellness, stress, and burnout. This methodology can be used in the future among other medical personnel to gain an in-depth understanding of specific stressors and coping mechanisms so that more focused wellness interventions and burnout mitigation measures can be implemented among these populations.

Our study also had notable limitations. The overall sample size was relatively small, and participants were all members of the same graduating class at one medical school. Therefore, the results represent the experiences of a limited group of students, and there is hence limited transferability of findings to other settings. Notably, numerous students did discuss the negative impacts of the COVID-19 pandemic, perhaps as a result of a recency effect; however, in addition to this, there was ample description by participants of a wide array of other stressors across the entire duration of medical school. Overall, the findings of this paper provide critical insights into the hardships and coping mechanisms faced by medical students throughout their years of studies.

These findings have the capability to inform programs and initiatives that seek to improve the overall well-being of medical students and to help them thrive; these insights can also guide programs in addressing the major issue of burnout. Given the variety of stressors faced by medical students and the different ways in which students cope, wellness initiatives could likely be improved by individualized strategies for students rather than a blanket approach for all.

## Conclusions

Burnout and its associated problems remain a major issue for medical students. Our study has provided some insight regarding the hardships faced, and means of coping, throughout the four years of medical school. It is hoped that these findings can be utilized by medical schools to help improve the overall well-being of medical students throughout their studies.

## Supplementary Information

Below is the link to the electronic supplementary material.

Supplementary file1 (DOCX 16 KB)

## Data Availability

Data is available upon reasonable request to authors.
