# Supplementary material for: Reflections of Graduating Medical Students: A Photo-Elicitation Study
Source: Med Sci Educ. 2023 Feb 16;33(2):363–7. doi: 10.1007/s40670-023-01758-3 (PMC9933809; doi:10.1007/s40670-023-01758-3)
Supplement: Supplementary file 1 — Supplementary file1 (DOCX 16 KB) [file 40670_2023_1758_MOESM1_ESM.docx]

**Supplementary Table 1: Codebook utilized for thematic analysis**

|  | Node/Code | Definition |
| --- | --- | --- |
| 1  [***a priori***](https://www.google.com/search?client=firefox-b-1-d&sxsrf=ALeKk01nIQDrjr80kC2FwRW-zFmtGawT7w:1589322911178&q=a+priori&spell=1&sa=X&ved=2ahUKEwiD8racsa_pAhWxTd8KHcN3BtQQBSgAegQIDRAn) | Stress | Use this code when respondent describes feeling overwhelmed, stresses, anxious, helpless or fearful (Do not use when respondents speak about others around them feeling stressed.) |
| 2  [***a priori***](https://www.google.com/search?client=firefox-b-1-d&sxsrf=ALeKk01nIQDrjr80kC2FwRW-zFmtGawT7w:1589322911178&q=a+priori&spell=1&sa=X&ved=2ahUKEwiD8racsa_pAhWxTd8KHcN3BtQQBSgAegQIDRAn) | Coping | Use this code when respondents describes how they cope, or strategies they use to cope |
| 3  [***a priori***](https://www.google.com/search?client=firefox-b-1-d&sxsrf=ALeKk01nIQDrjr80kC2FwRW-zFmtGawT7w:1589322911178&q=a+priori&spell=1&sa=X&ved=2ahUKEwiD8racsa_pAhWxTd8KHcN3BtQQBSgAegQIDRAn) | Interpersonal relationships | Use this code when respondent talks about interpersonal relationships (family, partner)- not colleagues/ classmates with whom respondent is “going through process with” etc.  Do not use for strain on interpersonal relationships if strain is due to unique life as a medical student (instead use “Identity not understood by others”) |
| 4  [***a priori***](https://www.google.com/search?client=firefox-b-1-d&sxsrf=ALeKk01nIQDrjr80kC2FwRW-zFmtGawT7w:1589322911178&q=a+priori&spell=1&sa=X&ved=2ahUKEwiD8racsa_pAhWxTd8KHcN3BtQQBSgAegQIDRAn) | Support | Use this code when respondent discusses sources of support, including friends, family, classmates, |
| 5  [***a priori***](https://www.google.com/search?client=firefox-b-1-d&sxsrf=ALeKk01nIQDrjr80kC2FwRW-zFmtGawT7w:1589322911178&q=a+priori&spell=1&sa=X&ved=2ahUKEwiD8racsa_pAhWxTd8KHcN3BtQQBSgAegQIDRAn) | Development of cynicism OR loss of idealism | Use this code when respondent speaks about negative or inappropriate attitudes toward patients, peers, or school/education; irritability, loss of idealism (eg loss of original motivations of helping others, contributing to greater good, etc), and withdrawal |
| 7 | Sleep/diet/exercise | Use this code when respondent discusses needs/short falls/strategies related to these items |
| 8 | Underpreparedness, gaps in education | Use this code when respondent expresses feelings of underpreparedness; also use this if respondent discusses lapses in education about things that relate to healthcare (eg public health) |
| 10 | Hierarchy | Use this code when respondent discusses chain of command in medical practice/ training or discusses medical school administration |
| 11 | Evaluation | Use this code when respondent describes SKMC examinations, evaluations, etc |
| 12 | Imposter Syndrome OR comparison with others | Use this code when respondent feels as though they are not qualified to be at present level of training; also use when someone compares themselves with others in the context of differing from how others cope with medical school, perform in medical school, etc |
| 13 | COVID-19 | Use this code when respondent discusses role of pandemic on med school experience |
| 14 | Board Exams | Use this code when respondent discusses STEP1/2 etc |
| 15 | Disconnected/ isolated | Use this code when respondent describes feeling removed from the outside world, or has lost identity of non-medical school roles; use when respondent reports feeling loneliness |
| 16 | On-hold / loss of interest in hobbies | Use this code when respondent discusses how their “rest” of life is on hold when you are in medical school; also use if respondent discusses loss of prior hobbies or interests |
| 17 | Camaraderie | Use this code when respondent describes benefits or positivity associated with working with other medical students |
| 18 | Uselessness or not knowing enough | Use this code when respondent describes their time or skills feeling worthless or unused in a medical school setting; also use when respondent describes feeling as though he/she/they does not know enough to be doing what he/she/they are doing |
| 19 | Competition | Use this code when respondent discusses comparison/ competition with others; use if someone uses word “competitive” |
| 20 | Anatomy | Use this code when respondent talks about Anatomy course at beginning of medical school |
| 21 | Compartmentalization | Use this code when respondent describes the need or experience of segmenting their roles (student, clinician) between multiple emotional experiences-- the need to “leave it at work” |
|  | Loss of identity/individualism | Use this code when a respondent discusses idea of feeling not like an individual, a “cog in the wheel;” also use when respondent discusses doing things mindlessly repetitively, “going through the motions”;  Do not use when a respondent discusses feeling separated or isolated from outside world |
|  | Identity Not understood (by others) | Use this code when respondent discusses feeling like their role or life as medical student is not understood by peers, friends, family, etc |
|  | Exhaustion | Use this when respondent describes feeling exhausted (emotionally or physically) |
|  | Extra-curricular activities | Use this when respondent describes coping with stress, pressure, etc with extra-curricular activities (other than exercise) |
|  | Development of substance use / unhealthy behaviors | Use this when respondent describes development of usage of substances such as recreational drugs, alcohol, or smoking, or any other self-described unhealthy behavior as a means of coping |
|  | Mental health treatment / therapy | Use this when respondent describes having mental health treatment / therapy |
